# Avoidance of Harm From Treatment for ANCA-Associated Vasculitis

**DOI:** 10.1007/s40674-017-0082-y

**Published:** 2017-11-13

**Authors:** Catherine King, Lorraine Harper

**Affiliations:** Centre for Translational Inflammation Research University of Birmingham Research Laboratories, Queen Elizabeth Hospital, Mindelsohn Way, Edgbaston, Birmingham, B15 2WB UK

**Keywords:** Anti-neutrophil cytoplasm antibody-associated vasculitides (AAV), Adverse events, Cyclophosphamide, Rituximab

## Abstract

*Purpose of review* With established immunosuppressant treatment regimens for anti-neutrophil cytoplasm antibody-associated vasculitides (AAV), prognosis has significantly improved. The mainstay of treatment still comprises high-dose corticosteroids and cyclophosphamide for severe forms, although rituximab is being increasingly utilised instead of cyclophosphamide as induction therapy. AAV patients experience an excess of infections, malignancies and cardiovascular events as compared to the general population, which is a combination of the systemic inflammatory process associated with vasculitis and the adverse events from treatment.

*Recent findings* Successful therapy should focus on suppressing disease activity and minimising treatment-related toxicity. Infection is the largest contributor to morbidity and mortality in the first year of treatment, and annual pneumococcal and influenza vaccinations, *Pneumocystis jiroveci* prophylaxis and tuberculosis (TB) and Hepatitis B virus screening are advised. Patients on high-dose corticosteroid treatment should have regular blood sugar monitoring, a FRAX assessment with vitamin D and calcium supplementation, consideration of prophylaxis for gastric ulcers and a cardiovascular risk assessment. Patients who are treated with cyclophosphamide could also receive MESNA to reduce the risk of chemical cystitis. Cyclophosphamide, methotrexate and azathioprine all require blood monitoring schedules due to the risk of bone marrow suppression, liver and renal toxicity. Hypogammaglobulinaemia is a recognised risk of rituximab treatment. Patients of reproductive age need to be counselled on the infertility risks with cyclophosphamide and the teratogenicity associated with it, methotrexate and mycophenolate mofetil.

*Summary* A greater focus on identifying clinical and biological markers that will help identify those patients at greatest risk of relapse, e.g. GPA and PR3-ANCA specificity, from those patients at greatest risk of toxicity, e.g. increasing age and declining GFR, is required to allow treatment to be tailored accordingly.

## Introduction

The introduction of effective immunosuppressive therapy to treat granulomatosis with polyangiitis (GPA) and microscopic polyangiitis, commonly grouped as anti-neutrophil cytoplasm antibody-associated vasculitis (AAV), has significantly changed prognosis. Untreated, these conditions are almost universally fatal [1]. Following the introduction of cyclophosphamide and corticosteroid-based immunosuppressive regimens, survival rates approach 80% at 5 years [2], with infection, cardiovascular disease and malignancy being the commonest causes of death. Although improved, this remains 2.6 times worse than an age and sex-matched healthy population, with death predicted by age, glomerular filtration rate < 15 ml/min and higher disease severity [2]. The greatest risk to patients with AAV is in the first year of diagnosis and most commonly from the adverse events associated with treatment rather than active vasculitis [3]. A study of patients with GPA using primary care data noted a 9-fold increase in mortality compared with a control population in the first year following diagnosis [4].

The improvement in survival has changed our perception of vasculitides from acutely fatal diseases to chronic conditions with a relapsing remitting nature and high morbidity due to disease damage and the side effects associated with treatment. There is a complex relationship between the severity of vasculitis and the risk of developing adverse events, with the estimated glomerular filtration rate (eGFR) being consistently found to be the greatest overall predictor. This is probably due to reduced excretion of active drug metabolites, as well as the general immunosuppressive effect of kidney failure. Damage due to disease and treatment is common and increases with time. In a long-term follow-up study of patients recruited to four European AAV trials, over 30% of patients reported greater than 5 damage items at long-term follow-up using the Vasculitis Damage Index (VDI) [5]. Patients with damage are also at increased risk of death [6].

The insight we now have into morbidity and mortality in AAV highlights the need for a change in our treatment approach to maximise efficacy and minimise toxicity.

## Treatment

### Treatment overview

The treatment of AAV is usually divided into two phases comprised of induction of remission and maintenance of remission. The latest 2015 recommendations from European league against rheumatism, European Renal Association and European Vasculitis Study (EULAR/ERA-EDTA) state that with organ or life-threatening disease, treatment typically involves pulsed cyclophosphamide or rituximab with high-dose corticosteroids (e.g. 1 mg/kg/day oral prednisolone, or intravenous methylprednisolone 0.5–1.0 g/day for 3 days) [7]. For those patients with pulmonary haemorrhage or rapidly progressive renal failure, plasma exchange should be considered. Patients with non-organ threatening disease may be successfully treated with a less toxic induction regimen of either methotrexate or mycophenolate mofetil alongside corticosteroids [7].

Maintenance therapy then consists of azathioprine, methotrexate or rituximab with corticosteroid tapering. There is great variation in the approach to corticosteroid tapering, but a target of between 7.5 and 10 mg of prednisolone (or equivalent) after 3 months of treatment is considered appropriate [7]. The duration of maintenance therapy is less clear.

Treatment must take into account quality of life (QOL) for patients with AAV. QOL among AAV patients is frequently poor and determined by multiple clinical and bio-psychosocial factors, of which fatigue appears to be of principal importance [8]. Whilst optimising disease control can improve QOL, non-pharmacological interventions targeting bio-psychosocial determinants may be more beneficial. AAV therapy must focus on person-centred outcomes such as QOL and ascertaining what a patient’s priorities are for the management of their chronic disease.

### Infection

Infection is a considerable cause of morbidity and mortality associated with AAV, and the immunosuppressive agents used to treat vasculitis are an undoubtable contributor to this. Overall, approximately 30% of patients will suffer from a serious infection requiring hospital admission, with respiratory infections being most common [9, 10]. The greatest risk to patients is in the first year, reflecting the intensity of immunosuppression [3, 11]. Higher doses of corticosteroids are also associated with infectious risk [9, 12, 13]. During infection, consideration should be given to pausing cytotoxic therapies especially if severe.

Advancing age and declining GFR are associated with increased risk of infection and mortality [3, 14, 15]. Immune dysfunction induced by AAV therapy predisposes to infection through neutropenia, reduced B and T cell numbers, aberrant cell function and reduced immunoglobulin levels [16]. Close monitoring of the peripheral blood count is recommended during AAV therapy as all cytotoxic therapies are associated with bone marrow suppression [3] (Table 1). Granulocyte colony-stimulating factor (G-CSF), commonly used in oncology for patients with neutropenia following chemotherapy, has been shown to be effective at reducing the duration of neutropenia in AAV and may be used safely, if necessary, without increasing risk of relapse [17].

**Table 1:** Treatment-specific blood monitoring schedules for ANCA vasculitis therapy (compiled from [29] and [55])

Drug	Laboratory monitoring	Frequency
Methotrexate	FBC LFTs, albumin Creatinine and eGFR	• Pre-treatment, baseline levels • Every 2 weeks until on stable dose for 6 weeks • Then once on a stable dose, monthly for 3 months • After this at least every 12 weeks • Dose increases should be monitored with FBC every 2 weeks until on stable dose for 6 weeks, then return to normal schedule • More frequent monitoring is required for patients at high risk for toxicity, i.e. chronic kidney disease and other risk factors for liver disease
Azathioprine	FBC LFTs, albumin Creatinine and eGFR	*as above
Pulsed cyclophosphamide	FBC LFTs, albumin Creatinine and eGFR	• Pre-treatment baseline levels • 7–10 days after first pulse • Then prior to giving next pulse • Unless dose changed, only require FBC monitoring prior to subsequent pulses • If WBC prior to pulse is < 4 × 10^9^/L and neutrophils < 2 × 10^9^/L, postpone treatment until above these counts and reduce next dose by 25%

Bacterial infections are common in AAV patients, accounting for up to 62% of major infections, in particular those causing pneumonia. Opportunistic *Pneumocystis jiroveci* pneumonia (PJP) has an incidence of 0.85–12% with increased mortality in AAV [18–20]. Although PJP is generally associated with a defect in T cell-mediated immunity, B cells have also been shown to play an important role in the defence against pneumocystis [21]. Prophylaxis with co-trimoxazole (800/160 mg on alternate days or 400/80 mg daily) is recommended for all patients undergoing cyclophosphamide or rituximab treatment [7].

GPA patients have higher *Staphylococcus aureus* colonisation of the upper respiratory tract which has been associated with a higher rate of relapse [22–24]. In patients with nasal disease, treatment with topical antibiotics such as mupirocin may be considered in the presence of chronic carriage of nasal *Staphylococcus aureus* [7, 25].

Vaccination should be used to reduce the risk of respiratory infections. The EULAR guidelines conclude that pneumococcal and seasonal influenza vaccinations are safe and effective and recommended for all patients with autoimmune inflammatory disease [11, 26]. Not all patients with AAV will have an adequate response to vaccination due to immune dysfunction [3]. Poor immunologic function, including low IgG, B cell counts and CD4 T cell counts, predict vaccine response and mortality [16]. Current EULAR recommendations are to avoid vaccination during B cell depletion therapy, e.g. with rituximab, as this has been consistently shown to impair humoral responses to influenza, pneumococcal and tetanus toxoid immunisations in patients with rheumatoid arthritis [27, 28]. Live attenuated vaccinations should be avoided in immunocompromised patients as live vaccines can, in some situations, cause severe or fatal infections due to extensive replication of the vaccine strain.

Mycobacterium tuberculosis infection has only rarely been reported in AAV patients, but can be fatal; the majority of cases show reactivation of latent TB. Current British Society of Rheumatology (BSR) and British Health Professionals in Rheumatology (BHPR) guidance recommends that all AAV patients require a thorough history, clinical examination and chest X-ray to screen for TB before commencing immunosuppressive therapy [25, 29]. TB prophylaxis is then indicated if latent TB is detected [25].

Viral infections are common, responsible for 35.8% of all major infectious episodes seen in one long-term analysis [9]. The occurrence of herpes zoster virus (HZV), shingles, occurs more often in AAV patients than a healthy population, with a frequency of 4.5 episodes of HZV/100 patient years compared to 0.40/100 patient years in the general population [12, 30]. Prophylaxis against varicella zoster virus (VZV) is not routinely recommended due to the non-life-threatening nature of VZV alongside the high risk of drug interactions and adverse events especially with renal dysfunction [25]. The varicella vaccine is a live attenuated vaccine and guidance for its use in immunosuppressed patients is mixed; if possible the vaccine should be administered prior to starting immunosuppression. Recently, a vaccine using recombinant VZV glycoprotein E has been shown to have benefit in reducing the risk of herpes zoster and post-herpetic neuralgia in those over 70 and would be suitable for use in immunosuppressed populations when available [31].

Latent infection with cytomegalovirus (CMV) is less frequent in AAV patients than in solid-organ transplantation, accounting for only 7.5% of major infectious episodes seen in long-term follow-up studies [9]. Whilst CMV polymerase chain reaction (PCR) monitoring and prophylaxis with valganciclovir are established in solid-organ transplantation follow-up, these are not routinely recommended in AAV [25].

Hepatitis B virus (HBV) reactivation occurs early in patients receiving rituximab or cyclophosphamide therapy for immune-mediated inflammatory disorders and is an important cause of hepatic failure [32]. All patients receiving significant immunosuppression should be screened for HBV infection and to perform HBV vaccination in patients with a negative result. Guidelines recommend that hepatitis B surface antigen (HBsAg)-positive patients receive preemptive antiviral therapy before starting therapy and that hepatitis B core antibody (HBcAb)-positive patients should be considered for preemptive antiviral therapy if rituximab or cyclophosphamide are given [32, 33].

Invasive fungal infections are rare in AAV patients, so systemic anti-fungal prophylaxis is not indicated. A Cochrane review noted that oral nystatin was no better than placebo at preventing candida infections in immunosuppressed patients. Consideration should be given to using fluconazole if prophylaxis or treatment is required [34].

Progressive multifocal leucoencephalopathy (PML) is a rare demyelinating infection of the central nervous system caused by John Cunningham virus which can result in irreversible neurological damage and death. Cases of PML have been reported in patients receiving cyclophosphamide and/or rituximab [35]. Clinicians must consider PML in their differentials for neurological presentations in patients who are heavily immunosuppressed as earlier diagnosis and restorations of immune function with withdrawal of immunosuppression may improve outcomes [36].

### Corticosteroids

Other adverse events are treatment specific. The extensive side effect profile associated with corticosteroids is well established. Data collected from four European trials showed that of AAV patients treated with corticosteroids within the first year, 8.2% developed new onset diabetes, 50% of which occurred within 1.7 months [3]. 29% of patients gained over 10 kg weight, 2.6% developed peptic ulceration, 2.5% had fractures, 2% developed cataracts and 0.4% developed avascular necrosis [3]. The adverse effects from corticosteroids are even more pronounced with increasing length of exposure. At long-term follow-up (median 5 years) of 270 patients, 41% had hypertension, 38% osteoporosis, 28% diabetes mellitus and 25% had developed cataracts [5].

Corticosteroid administration modulates carbohydrate metabolism particularly in adipose tissue as well as inducing insulin resistance, with impaired glucose uptake stimulating gluconeogenesis [37]. Risk factors for diabetes include previous corticosteroid-induced hyperglycaemia, pre-diabetes, obesity or a family history of diabetes [37]. Capillary blood glucose should be closely monitored whilst patients are on corticosteroids; if persistently over 12 mmol/L, treatment should be commenced.

Osteoporosis is a concern in patients with AAV. Corticosteroids promote osteoporosis through direct cellular effects stimulating a positive effect on osteoclasts, leading to increased bone resorption, and a negative effect on osteoblasts and osteocytes, causing reduced bone formation and reduced repair of micro-damaged bone. AAV alone is also associated with reduced BMD, although the mechanisms for this are not yet fully understood. In a cross-sectional study involving 99 patients with AAV treated with corticosteroids and cyclophosphamide, 78% of patients were found to have abnormal dual-energy X-ray absorptiometry (DEXA) measurements in one of three sites (the lumbar spine, proximal femur or radius); 21% of these patients had osteoporosis and 57% osteopenia [38]. Cumulative dosage of corticosteroid therapy was inversely related to Z-score; significant correlations were also seen between bone mineral density (BMD) and renal function [38]. Current UK guidelines developed by the National Osteoporosis Guideline Group (NOGG) advise assessment with FRAX® tool for all patients identified to be at risk of osteoporosis, to help guide further investigations and treatment.

Both the American College of Rheumatology and NOGG guidance suggests lifestyle advice should be given to all patients on commencement of long-term corticosteroid therapy including smoking cessation, alcohol limitation and regular exercise [39, 40]. Oral calcium and vitamin D supplementation is recommended for all patients undergoing long-term corticosteroid therapy [29, 41]. A Cochrane meta-analysis of 274 patients on corticosteroids taking calcium and vitamin D compared to placebo showed a clinically and statistically significant increase in bone mineral density of 2.6% at 2 years [41]. Bone-protective treatment with oral bisphosphonates should be started at the onset of corticosteroid therapy in individuals at moderate or high risk of fracture [40]. Bisphosphonate therapy is contraindicated in patients with significant renal impairment (GFR < 30 ml/min). Special consideration should be given to women of child-bearing potential due to the risk of foetal harm [40].

There is evidence that there is an increased risk of cardiovascular disease (CVD) in AAV patients [42]. Outcomes from the EUVAS trials showed that 14% of AAV patients have a cardiovascular event within 5 years of diagnosis; risk factors included MPO-ANCA positivity, increasing age and diastolic hypertension [43]. Whilst the mechanisms behind this association are not fully understood, it is believed that inflammation accelerates atherosclerosis through endothelial dysfunction [44]. Corticosteroid use may also increase cardiovascular risk [45]. Corticosteroids are known to cause multiple metabolic side effects including hypertension, hyperlipidaemia, weight gain and diabetes which are all significant cardiovascular risk factors. Due to this established increased risk of CVD in AAV patients, EULAR guidelines advise annual review of traditional Framingham risk factors [7].

It is also advisable to consider prophylactic gastric protection for AAV patients receiving a prolonged course of high-dose corticosteroids due to the risk of mucosal irritation.

Whilst it is appreciated that steroids are associated with significant toxicity, there is limited data available on the most appropriate duration of treatment or dose. Early withdrawal has been associated with increased disease relapse [46]. Data from EUVAS trials demonstrated that approximately half (47.8%) of the patients were on glucocorticoids beyond 2 years, with a mean duration of corticosteroid use of 40.4 months [5]. New studies are addressing the need and dose of corticosteroids required to treat AAV. PEXIVAS (plasma exchange and glucocorticoid dosing in the treatment of ANCA-associated vasculitis) addresses initial corticosteroid dosage (http://clinicaltrials.gov/ct2/ show/NCT00987389). The CLEAR (C5aR inhibitor on leukocytes exploratory ANCA-associated renal vasculitis) study has just been published investigating a new C5aR inhibitor avacopan as a replacement for high-dose glucocorticoids in treating newly diagnosed or relapsing AAV [47].

### Cyclophosphamide

Cyclophosphamide is an alkylating agent, which is metabolised by cytochrome P450 to acrolein and phosphoramide mustard. Phosphoramide mustard forms covalent bonds with DNA inducing lymphopenia, particularly of B cells and suppressing humoral responses. Cyclophosphamide metabolites are primarily excreted in the urine unchanged, resulting in increased systemic drug exposure in those with renal dysfunction. Dosing should be reduced for both age and renal function to reduce the risk of bone marrow suppression (Table 2). These metabolites are toxic to the bladder and can cause haemorrhagic cystitis and bladder cancer [48, 49]. Consideration should be given to the use of oral or intravenous 2-mercaptoethanesulfonate sodium (MESNA) for patients receiving intravenous cyclophosphamide alongside adequate hydration [7, 29]. MESNA binds to acrolein, a toxic metabolite of cyclophosphamide, rendering it non-toxic [49]. Current guidelines recommend routine surveillance with urinalysis; this should be continued indefinitely following initiation of any cyclophosphamide therapy. If non-glomerular haematuria is confirmed on urine microscopy, a urology referral should be sought [7].

**Table 2:** Dose modification of pulsed cyclophosphamide as used in the CYCLOPS trial and stated in the EULAR ANCA vasculitis guidelines [79]

Pulsed dose cyclophosphamide reductions for age and renal function		
Age (years)	Creatinine (μmol/L)	
	< 300	300–500
< 60	15 mg/kg/pulse	12.5 mg/kg/pulse
60–70	12 mg/kg/pulse	10 mg/kg/pulse
> 70	10 mg/kg/pulse	7.5 mg/kg/pulse

The cumulative dose of cyclophosphamide, with a threshold of 36 g, increases the risk of malignancy [50]. The overall incidence of cancer in AAV patients was previously reported as being up to two times higher than that of general population, with bladder cancer, non-melanoma skin cancer (NMSC), leukaemia and lymphoma being the most common malignancies [12, 14]. Recent studies suggest that with reduction in cyclophosphamide dosing, only NMSC is increased [51]. AAV patients should be educated on the risk of skin malignancy and should be strongly encouraged to attend the national cancer screening programs.

### Methotrexate

Methotrexate is an anti-folate drug with anti-proliferative and anti-inflammatory effects, due to the inhibition of several key enzymes involved in folate, methionine, adenosine and de novo nucleotide synthesis pathways [52]. Side effects are common with 10–37% of patients discontinuing treatment due to toxicity. GI toxicity is frequent although usually mild, associated with diarrhoea, nausea and vomiting; 30% of patients will develop abnormal LFTs although this maybe transient. Regular monitoring of liver function tests is recommended especially in those with other risk factors for liver disease [53] (Table 1). Patients are advised to limit alcohol intake whilst taking methotrexate to reduce the risk of liver toxicity.

Interstitial pneumonitis, although rare, remains a concern with methotrexate use; a low threshold for investigations is required for patients on methotrexate presenting with new or increasing dyspnoea or a dry cough. If detected early and methotrexate is discontinued, most patients recover without progression to lung fibrosis [53]. Renal failure from low-dose methotrexate is rare; however, as it is primarily excreted by the kidneys, it is contraindicated in severe renal impairment and should be used with caution if creatinine > 150 μmol/L.

All patients should be co-prescribed folic acid supplementation at a minimal dose of 5 mg once weekly, as adverse effects from methotrexate are thought to be mediated by folate antagonism [54, 55].

### Azathioprine

Azathioprine is a prodrug of mercaptopurine, which is subsequently metabolised by several pathways, one of which involves the enzyme thiopurine methyltransferase (TPMT). Azathioprine may cause a toxic reaction, which results in hepatotoxicity and myelosuppression and is related to variation in TPMT levels. Up to 14% of the population is known to have reduced levels of this enzyme as a result of genetic mutations [56]. The positive predictive value of measuring TPMT levels is widely debated, but British Society of Rheumatology guidelines state that patients should have baseline TPMT status assessed [55]. Allopurinol, a xanthine oxidase inhibitor, an enzyme involved in metabolising 6-mercaptopurine, should not be used with azathioprine due to the high risk of bone marrow suppression.

### Rituximab

The safety of rituximab has become a topic of great interest with its increasing use in both remission induction and maintenance therapy in AAV. Rituximab was first used successfully to treat rheumatological disease without significant rates of infection at long-term follow-up, so its promise in reducing the toxicity from cumulative doses of cyclophosphamide and ongoing maintenance therapy was particularly appealing [57, 58]. Whilst its efficacy has been demonstrated, its side effect profile remains in question as the trials demonstrating efficacy failed to show any difference in adverse events, such as infection, in patients with AAV [59, 60].

The advantages of rituximab in reducing infections rates as demonstrated in patients with rheumatoid arthritis, may not have materialised within these trials as the follow-up periods may not have been long enough to have incorporated the benefit from reduced cumulative cyclophosphamide exposure and prolonged maintenance therapy [60].

Hypoimmunoglobulinaemia is associated with the use of cyclophosphamide and rituximab [61]. A recent retrospective study of 243 patients with multi-system disease treated with rituximab demonstrated that 56% had IgG hypoimmunoglobulinaemia during follow-up; although in 50%, this was transient. Only 4.2% of these patients required serum IgG replacement for infection [62]. EULAR guidelines state that surveying patients with AAV is warranted post cyclophosphamide and rituximab treatment for serum immunoglobulin concentrations and persisting hypoimmunoglobulinaemia [7].

Late-onset neutropenia (LON), defined as an absolute neutrophil count (ANC) < 1.5 × 10^9^/L at least 4 weeks after the last infusion, has been observed in patients with rheumatological disease, including AAV treated with rituximab [63]. A recent study involving 59 patients with AAV treated with rituximab demonstrates that 7 patients (11.9%) developed LON after a median time of 86 days (range 56–168 days) since their latest rituximab treatment [64]. LON is idiosyncratic in its development; it may occur after both single and repeated rituximab courses and does not recur after subsequent doses of rituximab. No predisposing factors for LON have been identified.

### Fertility and pregnancy

Disease activity and its therapy are threats to the fertility of patients with vasculitis. The cumulative dose of cyclophosphamide and increasing age at initiation of treatment both increase the risk of primary ovarian failure [65, 66]. Data from a lupus trial suggests that a total cyclophosphamide exposure of 14–20 g causes infertility in > 50% of women aged over 32 years, with a lower risk in younger women [67]. The long-term effects of rituximab on fertility and teratogenicity during pregnancy have not been studied, but no concerns have been reported suggesting rituximab should be used for young patients with AAV wishing to preserve fertility [7].

Cyclophosphamide, methotrexate and mycophenolate mofetil are teratogenic. Patients taking these agents should be counselled on this and advised to use effective contraception for the duration of therapy and at least 3 months thereafter. Azathioprine is considered low risk in pregnancy without a significant increase in major congenital malformations [68, 69]. There is less guidance available on the risk of paternal immunosuppressant exposure. The British Association of Dermatologists’ guidelines for the use of azathioprine state that studies do confirm male patients receiving azathioprine father healthy children and standard dose azathioprine does not appear to affect fertility [70].

There is still little research on pregnancy outcomes in patients with vasculitis. Two recent reports suggest that relapse during pregnancy is uncommon and that outcomes when disease is controlled prior to conception are good [71, 72].

### Thromboembolic disease

Although microthrombosis is a component of vasculitic pathology, the frequency of deep venous thrombosis and pulmonary embolism is also increased, being highest during periods of disease activity and inflammation. A rate of 15% was reported in a prospective trial; and in retrospective surveys, rates of 1.8%/year for all AAV patients rising to 6.7–9%/year for those with active disease were reported [73]. Autoantibodies to plasminogen and tissue plasminogen activator occur in the sera of 25 and 14% of AAV patients [74]. Their presence has been associated with more severe renal outcomes and increased risk for thromboembolic events. Anti-coagulants have been used historically in the treatment of renal vasculitis, but thromboprophylactic strategies have not been developed for AAV in the current era. Until further data emerges of the clinical utility of anti-plasminogen autoantibody testing, it is advisable to address conventional risk factors and maintain a high threshold for suspicion of thromboembolism.

### Risk of relapse versus prolonged maintenance therapy

Relapse in AAV occurs in 30–50% of patients by 5 years, with an increased risk associated with GPA, PR3-ANCA specificity, ear nose and throat involvement, persisting ANCA positivity after induction therapy, a lower serum creatinine at diagnosis and early withdrawal of corticosteroids or immunosuppressives [75]. However, prolonged duration of maintenance therapy is associated with increased exposure to the toxicities of immunosuppressives and corticosteroids. Current recommendations suggest at least 24 months of induction therapy once remission has been obtained, although, the optimal duration of remission-maintenance therapy remains unknown [7].

The recent REMAIN trial (prolonged REmission-MAINtenance therapy in systemic vasculitis) by EUVAS looked at whether continued azathioprine/prednisolone was more effective in preventing relapse in AAV than their withdrawal at 24 months. It demonstrated that continuation of corticosteroids and azathioprine beyond 2 years reduced the relapse risk and improved renal outcome. Continuation of immunosuppressive therapy was associated with a trend towards more frequent adverse events such as malignancy, infections and cardiovascular complications (*p* = 0.07) [76]. However, another study investigating the use of prolonged azathioprine to prevent disease relapse failed to show benefit [77].

Further research is required to identify clinical or biological markers that will help to identify high-risk patients who require prolonged therapy, until then, the treating clinician must weigh up the individual risk of relapse compared with the risk of treatment toxicity.

## Conclusion

Over the last 20 years, careful use and monitoring of cyclophosphamide and corticosteroid-based treatment regimens have improved outcomes [78]. This may be improved further with use of rituximab as an alternative to cyclophosphamide and new therapies reducing the need for corticosteroids.

It is the responsibility of the treating clinician to assess every patient as an individual, considering the risks associated with AAV therapy before commencement of induction therapy, using the risk factors identified for adverse events and mortality including increasing age and declining kidney function [3]. Individual patient risk of relapse must be balanced against risk from treatment toxicity when deciding duration of maintenance therapy.

The impact of this chronic relapsing disease and the burden of therapy on patients’ physical and emotional wellbeing are significant, with fatigue being a considerable concern. Many patients experience reduced quality of life despite successful therapy. We must identify patient priorities at the start of treatment with regular reassessment, alongside patient education on both the nature of their disease and the considerable risks from disease and therapy. This will aid clinicians and patients with shared decision-making to achieve the best outcomes for patients with AAV.

Until the development of treatments without the significant side effects that still remain from current regimens, we need to focus on prophylaxis to prevent the known toxicity from AAV therapies.
